# Patients with anorectal malformation and upper limb anomalies: genetic evaluation is warranted

**DOI:** 10.1007/s00431-015-2655-9

**Published:** 2015-10-24

**Authors:** Desiree van den Hondel, Charlotte H. W. Wijers, Yolande van Bever, Annelies de Klein, Carlo L. M. Marcelis, Ivo de Blaauw, Cornelius E. J. Sloots, Hanneke IJsselstijn

**Affiliations:** Department of Pediatric Surgery, Erasmus MC–Sophia Children’s Hospital, Room SK-1280, P.O. Box 2060, 3000 CB Rotterdam, The Netherlands; Department for Health Evidence, Radboud University Medical Center, Nijmegen, The Netherlands; Department of Clinical Genetics, Erasmus MC, Rotterdam, The Netherlands; Department of Human Genetics, Radboud University Medical Center, Nijmegen, The Netherlands; Department of Surgery-Pediatric Surgery, Amalia Children’s Hospital, Radboudumc, Nijmegen, The Netherlands

**Keywords:** Anorectal malformation, Anorectal atresia, Upper extremity deformities, congenital, Syndrome, VACTERL association

## Abstract

The objective of this study was to compare the prevalence of genetic disorders in anorectal malformation (ARM) patients with upper limb anomalies to that in ARM patients with other associated anomalies. A retrospective case study was performed in two pediatric surgery centers. All patients born between 1990 and 2012 were included. VACTERL (vertebral defects (V), anal atresia (A), cardiac malformations (C), tracheoesophageal fistula with esophageal atresia (TE), renal dysplasia (R), and limb anomalies (L)) was defined as at least three components present. We included 700 ARM patients: 219 patients (31 %) had isolated ARM, 43 patients (6 %) had a major upper limb anomaly, and 438 patients (63 %) had other associated anomalies. The most prevalent upper limb anomalies were radial dysplasia (*n* = 12) and hypoplastic thumb (*n* = 11). Ten of the 43 patients (23 %) with an upper limb anomaly were diagnosed with a genetic disorder—nine also met the VACTERL criteria—vs. 9 % of ARM patients with other anomalies (*p* = 0.004, chi-squared test).

*Conclusion*: Genetic disorders are twice as frequently diagnosed in ARM patients with upper limb anomalies than in those with other anomalies. As they also frequently meet the VACTERL criteria, it is important to consider VACTERL as a diagnosis per exclusionem. Genetic counseling is certainly warranted in these patients.

**Table:** 

**What is Known**: • *Anorectal malformations* (*ARMs*) *often co-occur with other congenital anomalies*, *including upper limb anomalies*, *mainly of pre-axial origin*. • *Co-occurrence of ARMs and upper limb anomalies is seen in disorders such as Townes-Brocks syndrome*, *Fanconi anemia*, *and VACTERL association*.
**What is New**: • *ARM patients with a major upper limb anomaly*—*with or without other congenital anomalies*—*have a twofold greater chance of a genetic disorder than have non-isolated ARM patients without upper limb anomalies*. • *Not all upper limb anomalies in ARM patients are part of the VACTERL association*; *a workup for genetic evaluation is proposed*.

**Electronic supplementary material:**

The online version of this article (doi:10.1007/s00431-015-2655-9) contains supplementary material, which is available to authorized users.

## Introduction

Anorectal malformations (ARMs) are rare congenital anomalies that occur in approximately 1 to 3 in every 5000 live births [13]. Of the ARM patients, 43 to 71 % have additional congenital anatomical anomalies [3, 6, 11, 15, 27]. These include a great variety of upper limb anomalies, from a mild hypoplastic thumb to severe radial dysplasia [7, 10, 11, 18–20, 26, 29].

Some types of upper limb anomalies are associated with specific syndromes. For example, thumb anomalies may indicate Townes-Brocks syndrome, given the fact that 89 % of the patients with Townes-Brocks syndrome have a thumb anomaly [16], or they may even indicate Fanconi anemia (prevalence of thumb anomalies 50 % [28]). Ulnar deficiencies may be suggestive of, for example, ulnar-mammary syndrome [4]. Once evaluation has excluded known syndromes, VACTERL association can be considered, which refers to vertebral defects (V), anal atresia (A), cardiac malformations (C), tracheoesophageal fistula with esophageal atresia (TE), renal dysplasia (R), and limb anomalies (L). VACTERL association is mainly associated with preaxial limb defects [5].

Naturally, patients with more than one congenital anatomical anomaly are more likely to be diagnosed with a syndrome than are patients with a single congenital anomaly. However, in our experience, ARM patients with an upper limb anomaly—with or without other congenital anomalies—are more frequently diagnosed with a syndrome than are non-isolated ARM patients without upper limb anomalies. The aim of this study was to answer the following questions:

1. What is the prevalence of upper limb anomalies in ARM patients?
2. What upper limb anomalies are most frequently seen in ARM patients?
3. Are syndromes more prevalent in ARM patients with a major upper limb anomaly—with or without other additional congenital anomalies—compared to non-isolated ARM patients without an upper limb anomaly?

## Materials and methods

### Study sample

A retrospective case study was performed on all patients with an ARM born between 1 January 1990 and 1 July 2012 and treated in one of the participating university pediatric surgery centers (Erasmus MC-Sophia Children’s Hospital, Rotterdam, the Netherlands, and Amalia Children’s Hospital, Radboudumc, Nijmegen, the Netherlands). Patient characteristics were obtained from the medical records, with special attention to the presence of upper limb anomalies. This study was approved by the Erasmus MC Medical Ethical Review Board.

**Fig. 1:**
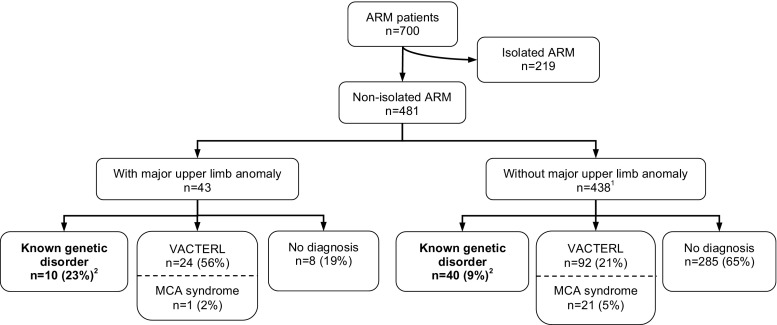
Flow chart of patient selection. *ARM* anorectal malformations, *MCA syndrome* multiple congenital anomalies syndrome. VACTERL was defined as three or more components present. *Superscript number 1*, including 15 patients with a minor upper limb anomaly. As the minor anomalies, as classified by the clinical geneticists, are subjective anomalies; these were not included in the main analysis. *Superscript number 2* represents *p* = 0.004, chi-squared test. Isolated ARM patients were excluded from analysis. The patients who had an upper limb anomaly were significantly more frequently diagnosed with a genetic disorder than those with other associated anomalies

Two main groups were distinguished: isolated ARM patients and non-isolated ARM patients. The latter group was subdivided into patients with and without major upper limb anomalies (Fig. 1). The prevalence of genetic disorders was determined in these two subgroups.

### Classification systems

ARMs were classified by the Krickenbeck classification [12]. VACTERL association was considered to be present if three or more components of the acronym were identified [21, 25]. In our centers, all patients with ARM are screened for VACTERL association as follows: X-rays of the spine (V), echocardiogram of the heart (C), X-ray of the chest after insertion of a nasogastric tube (TE), ultrasound of the abdomen (R), and physical examination of the limbs (L).

Upper limb anomalies were classified as major or minor by the clinical geneticists (YB and CM). Examples of major anomalies are radial or ulnar dysplasia and polydactyly, and of minor anomalies are single palmar crease, long fingers, or long, coarse hands. As the minor anomalies are subjective anomalies, especially since this is a retrospective study, these were not included in the main analysis but mentioned separately.

Most major upper limb anomalies had been classified by the plastic surgeon as part of regular care (radial dysplasia and thumb hypoplasia were classified according to James et al. [14] and Abdel-Ghani et al. [1], respectively). Polydactyly was classified as preaxial or postaxial. Major upper limb anomalies in patients who had not been seen by the plastic surgeon were classified as type unknown (*n* = 4) and described according to the medical charts.

Patients with multiple congenital anomalies who had a pure clinical diagnosis, for which the underlying genetic defect is unknown, such as Goldenhar syndrome, were classified as “multiple congenital anomalies (MCA) syndrome” and not as “genetic disorder.”

### Statistical analysis

Results were shown as number (%) or as median (range). Continuous variables were compared using the Mann-Whitney *U* test, whereas proportions were compared using the chi-squared test.

The prevalence of genetic disorders was compared between ARM patients with a major upper limb anomaly—with or without other associated congenital anomalies—and non-isolated ARM patients without an upper limb anomaly. This was also done for organ systems, being lower limb, cardiac, central nervous system (CNS), urogenital, other gastrointestinal, and vertebral anomalies. Further, Phi analysis was conducted to determine whether anomalies in different organ systems were associated with each other.

## Results

In total, 219 of the evaluated 700 patients (31 %) had an isolated ARM and were excluded from further analyses (Fig. 1). A major upper limb anomaly had been documented in 43 patients (6 %). Radial dysplasia was the most prevalent major upper limb anomaly (*n* = 12; 28 %), followed by thumb hypoplasia (*n* = 11; 26 %). Table 1 provides details of all major upper limb anomalies. Four patients were not classified by the plastic surgeon and were therefore classified as type unknown. The remaining 438 patients all had an ARM with other associated anomalies.

**Table 1:** Detailed findings in 43 anorectal malformation patients with a major upper limb anomaly

		Additional description	Disorder
Radial dysplasia; *n* = 12			
Type 0	Unilateral	With thumb hypoplasia type 3	VACTERL (trisomy X)
	Bilateral	With thumb hypoplasia type 2	22q11 microduplication (maternal)
Type 2	Unilateral	With thumb hypoplasia type 4; other hand radial dysplasia type 1 with thumb hypoplasia type 1	Goldenhar syndrome
	Unilateral	Unilateral radial dysplasia type 2	–^a^
	Bilateral	With thumb hypoplasia type 4	VACTERL
Type 4	Unilateral	With micromelia of 3 digits	VACTERL^b^
	Unilateral	With thumb hypoplasia type 5 and syndactyly 2nd and 3rd digit; other hand thumb hypoplasia type unknown	VACTERL^b^
	Unilateral	Other hand radial dysplasia type 1	VACTERL
	Bilateral	Bilateral radial dysplasia type 4	VACTERL
	Bilateral	With thumb hypoplasia type 5	VACTERL
	Bilateral	With thumb hypoplasia type 5; syndactyly 2nd and 3rd digit, hypoplasia 2nd digit, camptodactyly all digits	Fanconi anemia (no mutation known)
Type unknown	Bilateral	Bilateral radial dysplasia, type unknown	Trisomy 18^b^
Thumb hypoplasia without apparent radius involvement; *n* = 11			
Type 2	Unilateral	Unilateral thumb hypoplasia type 2	VACTERL^a^
	Unilateral	Unilateral thumb hypoplasia type 2	VACTERL^c^
	Unilateral	Unilateral thumb hypoplasia type 2	VACTERL
	Unilateral	Other hand thumb hypoplasia type 1	VACTERL
Type 3	Unilateral	Unilateral thumb hypoplasia type 3	VACTERL^b^
	Bilateral	One hand triphalangeal thumb	VACTERL
Type 4	Bilateral	Bilateral thumb hypoplasia type 4	VACTERL^b^
Type 5	Unilateral	Other hand thumb hypoplasia type 2	VACTERL
	Unilateral	Other hand thumb hypoplasia type unknown	VACTERL^b^
Type unknown	Unilateral	Unilateral thumb hypoplasia type unknown	(16q12.1) polymorphism
	Unilateral	Unilateral thumb hypoplasia type unknown	–^a^
Ulnar dysplasia; *n* = 1			
	Unilateral	Unilateral longitudinal ulnar growth arrest (1 thumb, 3 digits, floating 4th digit). Other hand cleft hand between 4th and 5th digit	Ulnar-mammary syndrome, heterozygous *TBX3* mutation (maternal)
Preaxial polydactyly; *n* = 5			
	Unilateral	Extra thumb	VACTERL
	Unilateral	Extra thumb	VACTERL
	Bilateral	Extra thumb	^a^
	Bilateral	Extra thumb	^a^
	Bilateral	One hand extra thumb, other hand 7 digits; both hands syndactyly thumb and 2nd digit and triphalangeal thumb	Townes-Brocks syndrome, *SALL1* mutation
Postaxial polydactyly; *n* = 2			
	Bilateral	6 fingers; bilateral camptodactyly 2nd–4th digit	Trisomy 13^b^
	Bilateral	Extra 6th metacarpal	–^a^
Thumb hyperplasia; *n* = 2			
	Unilateral	Same hand single palmar crease	VACTERL
	Unilateral	Both hands also clasped thumb	VACTERL
Other; *n* = 10			
	Unilateral	Triphalangeal thumb	Blackfan-Diamond anemia (no mutation known)
	Bilateral	Syndactyly 3rd–5th digit; other hand syndactyly 3rd–4th digit	Trisomy 21
	Bilateral	Syndactyly 3rd–5th digit; other hand absence of 5th digit; bilateral nail dysplasia	VACTERL^b,d^
	Unilateral	Brachymesophalangy 5th digit	–^a^
	Unilateral	Clasped thumb	–^a^
	Bilateral	Clasped hands	VACTERL
	Unilateral	Deviating implantation of the thumb	VACTERL^a^
	Bilateral	Deviating implantation of the thumb	VACTERL
	Unknown	Clino/brachydactyly not further specified	del(1)(q23q25)^b^
	Bilateral	Clubbing hand, long fingers	Cri du Chat syndrome, der(5).t(5;14)^b^

^a^No known genetic disorder, does not meet criteria VACTERL association

^b^Patient deceased

^c^S*ALL-1* mutation (Townes-Brocks syndrome) still needs to be excluded

^d^ *GLI-3* mutation (Pallister Hall syndrome) still needs to be excluded

In 10/43 patients who had a major upper limb anomaly—with or without other congenital anomalies—a genetic disorder had been diagnosed (23 %; Table 2). Nine of these patients also met the criteria of VACTERL association. Of the remaining 33/43 patients, 24 had been diagnosed with VACTERL association per exclusionem (that is, 56 % of patients with major upper limb anomaly) and one with Goldenhar syndrome; of the other eight patients, four had additional congenital anomalies but did not meet the VACTERL criteria and four had no other anomalies besides the ARM and upper limb anomaly.

**Table 2:** Details of 10 patients with a major upper limb anomaly and a genetic disorder

Genetic anomaly	Disorder (OMIM)	Associated anomalies
Numerical chromosomal disorders		
Trisomy 13^a^	Patau syndrome	Typical dysmorphic features, possible esophageal atresia, ASD, VSD, overriding aorta, absent external auditory canal, micropenis, non-descended testes
Trisomy 18^a^	Edward syndrome	Typical dysmorphic features, VSD, intrauterine growth retardation
Trisomy 21	Down syndrome	Typical dysmorphic features
Microdeletions/duplications		
22q11 duplication	22q11 microduplication syndrome (#608363)	Kidney agenesis, caudal regression syndrome, esophageal atresia, VSD; mother had same duplication
Heterozygous mutation *TBX3*	Ulnar-mammary syndrome (#181450)	Congenital subglottic stenosis, ASD, non-descended testes, mother had same mutation
*SALL 1* mutation	Townes-Brocks syndrome (#107480)	Bilateral dysplastic kidneys, hemivertebrae, club foot, hearing loss
Diagnosis confirmed by hematologic investigations	Blackfan-Diamond anemia (#105650)	VSD
del(1)(q23q25)^a^	No reference available	Dysmorphic features, kidney agenesis, dextrocardia, esophageal atresia, abnormal hearing
Diagnosis confirmed by chromosomal breakage tests^b^	Fanconi anemia	Esophageal atresia, ADS, open ductus Botalli, hypospadia, hearing loss, non-descended testes; familial
der(5)t(5;14) ^a^	Cri du Chat syndrome (#123450)	Dysmorphic features, VSD, bicuspid aortic valve, uterus didelphys, enlarged kidney

*OMIM*, Online Mendelian Inheritance in Man, *ASD* atrial septal defect, *VSD* ventricular septal defect, *AVSD* atrioventricular septal defect

^a^Patient deceased. Due to treatment withdrawal, these patients were not all fully screened for other congenital anomalies

^b^Parents did not consent for mutation analysis

Forty of the 438 patients with other associated anomalies (9 %) had been diagnosed with a genetic disorder: 23 (5 % of patients with non-isolated ARM without a major upper limb anomaly) with a numerical chromosomal disorder (mostly trisomy 21, *n* = 17; others were Turner syndrome and trisomy X) and 17 (4 %) with a microdeletion or duplication. Of these 40 patients, 14 (35 %) met the criteria for VACTERL association. Of the 398 remaining patients, 21 were diagnosed with an MCA syndrome and 92 patients met the criteria for VACTERL association.

The prevalence of genetic disorders (thus excluding VACTERL association and MCA syndromes) in the group of ARM patients with a major upper limb anomaly—with or without other anomalies—was significantly higher than that in the group of ARM patients with other associated anomalies: 23 vs. 9 %, respectively; *p* = 0.004, chi-squared test.

The patient characteristics of the non-isolated ARM patients with and without major upper limb anomalies are shown in Table 3. The prevalence of urogenital anomalies did not differ between both groups (60 and 62 %), but cardiac anomalies (varying from atrial or ventricular septal defect to coarctation of the aorta or Fallot tetralogy) occurred more frequently in the patients with a major upper limb anomaly than those without (60 and 33 %, respectively; *p* < 0.001). The same was true for gastrointestinal anomalies (44 vs. 18 %, respectively; *p* < 0.001). The most common gastrointestinal anomaly was esophageal atresia (with or without fistula), occurring in 13 patients of the major upper limb anomaly group (30 %). Others were duodenal atresia, small bowel atresia, and choledochal cyst.

**Table 3:** Background characteristics of non-isolated anorectal malformation patients

	Non-isolated ARM with major upper limb anomaly	Non-isolated ARM without upper limb anomaly
Number of patients	*n* = 43	*n* = 438
Type of ARM		
Perineal fistula	12 (28 %)	139 (32 %)
Rectourethral fistula		
Bulbar	2 (5 %)	31 (7 %)
Prostatic	5 (12 %)	46 (11 %)
Unknown	5 (12 %)	38 (9 %)
Rectovesical fistula	0	15 (3 %)
Vestibular fistula	7 (16 %)	62 (14 %)
Cloaca	3 (7 %)	34 (8 %)
Other	2 (5 %)	55 (13 %)
Unknown	7 (16 %)	18 (4 %)
Male sex	22 (51 %)	276 (63 %)
Gestational age (weeks)	38.5 (29–43)	38.0 (26–42)
Birth weight (g)	2610 (1020–3920)	2980 (625–4760)
Associated anomalies^a^		
Urogenital	26 (60 %)	272 (62 %)
Cardiac	26 (60 %)*	145 (33 %)*
Other skeletal^b^	20 (47 %)	202 (46 %)
Gastrointestinal	19 (44 %)*	77 (18 %)*
Central nervous system	8 (19 %)	96 (22 %)
Pulmonary	4 (9 %)	28 (6 %)

*ARM* anorectal malformation

Results are presented as *n* (%) or as median (range)

*p < 0.001, chi-squared test

^a^Several patients had more than 1 associated anomaly

^b^Skeletal anomalies other than upper limb anomalies

We included 15 patients with a minor upper limb anomaly in the non-upper limb anomaly group. These minor anomalies were single palmar crease, long fingers, or large, coarse hands. Five of these patients (33 %) had been diagnosed with a genetic disorder: two patients had trisomy 21, one had 47,XY,+der(22) (Cat eye syndrome; OMIM #607575), one had deletion 17p13.3 (Miller-Dieker syndrome; OMIM #247200), and one had duplication of chromosome band 3p12.2 (no reference available).

The prevalence of genetic disorders in patients with associated anomalies other than upper limb anomalies is shown in Online Supplemental Table 4. The associations between the different associated anomalies were all poor (Online Supplemental Table 5).

## Discussion

The prevalence of major upper limb anomalies in this cohort of 700 ARM patients was 6 %. The most prevalent anomalies were radial dysplasia and thumb hypoplasia. Of the patients with a major upper limb anomaly, 23 % had a genetic disorder versus 9 % of other non-isolated ARM patients.

An extensive literature search yielded eight publications describing the prevalence of upper limb anomalies in ARM patients [7, 10, 11, 18–20, 26, 29]. The prevalence ranged from 2 to 12 % in study cohorts varying from 99 to 1417 ARM patients, which is in concurrence with the present study. However, none of these studies provided details of types of anomalies or numbers of syndromes diagnosed in this patient group. In present study, we found that the most prevalent upper limb anomalies were radial dysplasia and thumb hypoplasia.

Unfortunately, because of the retrospective nature of this study, we were unable to determine at what ages the upper limb anomalies had been diagnosed. The prevalence of subtle upper limb anomalies such as thumb hypoplasia—in our study sample one of the most prevalent upper limb anomalies—could very well be underestimated in newborns. It might not only be because of the mere size of the hand and thumb but also when, for example, a plaster for a peripheral intravenous line covers the hand. Early recognition of the thumb anomaly is not only wanted for optimal treatment of the thumb [1, 17] but also even more for early recognition of syndromes such as Fanconi anemia. Even though Fanconi anemia is a rare disorder, the clinical consequences for the child warrant early recognition and this will also permit appropriate counseling of the parents. Unfortunately, we were unable to determine whether Fanconi syndrome had been excluded in all patients with radial limb deficiencies, but chromosomal breakage tests were available in our country from the beginning of the study period.

Furthermore, the prevalence of other genetic diagnoses may have been underestimated because the possibilities for genetic testing have advanced rapidly since 1990. In the 1990s, diagnostic investigations in medical genetics mostly consisted of karyotyping. In the Netherlands, Townes-Brocks syndrome could be confirmed by Sanger sequencing from the late 1990s and 2005, respectively. Nowadays, the widespread application of affordable microarray approaches provides improved screening for genetic disorders [2]. In the Netherlands, clinical geneticists use arrays for screening since 2011. Further improvements in genetic diagnostics are to be expected with next-generation sequencing, where even smaller events can be detected and an absolute copy number prediction is possible [2, 23].

It is important to consider VACTERL association as a diagnosis per exclusionem and to have a keen eye for specific genetic disorders in this patient group, not only for the clinical consequences for the child but also to adequately counsel the parents in the case of an inheritable disorder. Ten of the 43 patients (23 %) with a major upper limb anomaly were diagnosed with a genetic disorder. This prevalence is higher than in patients without a major upper limb anomaly (9 %) and also higher than reported in patients with an upper limb anomaly without an ARM (7–17 %) [8, 9].

Besides the major upper limb anomalies, 15 patients had a minor upper limb anomaly, mostly large, coarse hands or deviating implant of the thumb. Compared to the major limb anomalies, a larger proportion of this group was diagnosed with a genetic disorder (33 %). This might be biased, as clinicians may tend to more actively search for small anomalies when a disorder is suspected based on dysmorphic features or a specific pattern of congenital anomalies, compared to the situation in which there is an isolated ARM without apparent associated anomalies. However, we recommend to consult a geneticist specialized in dysmorphology in all ARM patients, as these minor features can be hard to recognize while they still might hint towards a specific genetic disorder.

Cardiac and gastrointestinal anomalies were documented almost twice as much in patients with a major upper limb anomaly compared to other non-isolated ARM patients. Still, Phi analysis showed only poor associations between these anomalies and upper limb anomalies. These anomalies in part are inherent to the syndromes these patients were diagnosed with, but they can also be part of VACTERL association [22]. VACTERL screening in the neonatal period is internationally recommended for all ARM patients [24]. Preoperative cardiac screening by ultrasound should strongly be considered especially in ARM patients with an upper limb anomaly in order to minimize anesthesiologic risks.

**Fig. 2:**
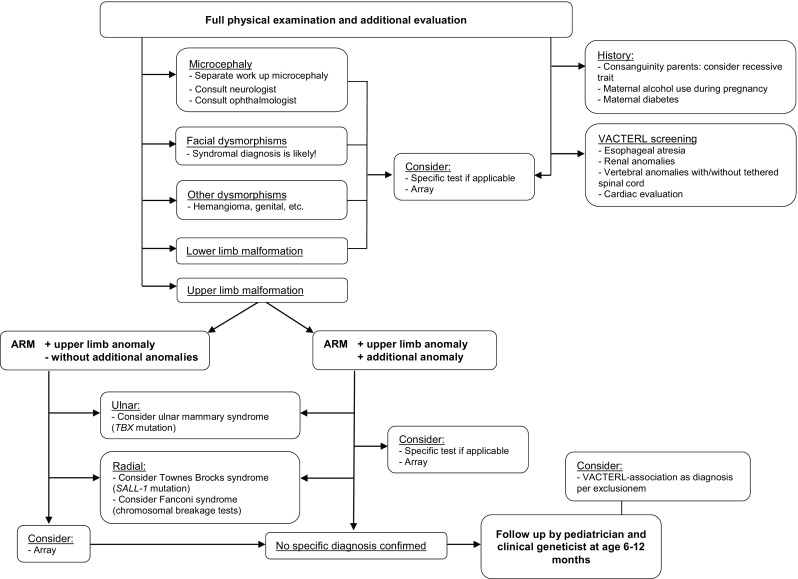
Algorithm for workup of a patient with an anorectal malformation, with special attention to upper limb anomalies. *ARM* anorectal malformation. In this algorithm, which focuses on a workup for anorectal malformation patients with an upper limb anomaly, only the most relevant syndromes are mentioned. The purpose of this algorithm is to provide a general approach for pediatricians; it does not provide a complete genetic overview. This algorithm was designed based on current literature and experience of clinical geneticists, and on implementation of the findings of this study into clinical practice

This study provides new insights for the workup of a neonate with an ARM. When an upper limb anomaly is present, the pediatrician should be alert to genetic disorders. We propose an algorithm for genetic workup for ARM patients (Fig. 2). This algorithm includes the most relevant syndromes in order to provide a general approach. A clinical geneticist specialized in dysmorphology should be counseled when an ARM co-occurs with an upper limb anomaly, because of the great diversity of genetic disorders present in this patient group.

Concluding, the prevalence of major upper limb anomalies in ARM patients is 6 %. The most frequent anomalies were radial dysplasia and thumb hypoplasia. ARM patients with a major upper limb anomaly are twice as frequently diagnosed with a genetic disorder compared to ARM patients with other associated anomalies. Ninety percent of patients with a major upper limb anomaly and genetic disorder met the criteria for VACTERL association; it is therefore important to consider VACTERL association as a diagnosis per exclusionem and to be conscious of genetic disorders in patients with ARM and an upper limb anomaly. Consultations by a clinical geneticist specialized in dysmorphology in all ARM patients could help optimize screening for other syndromes.

## Electronic supplementary material

ESM 1 (DOC 68 kb)

## Abbreviations

ARM: Anorectal malformations
CNS: Central nervous system
MCA syndrome: Multiple congenital anomalies syndrome
OMIM: OMIM, Online Mendelian Inheritance in Man
VACTERL association: Vertebral defects (V), anal atresia (A), cardiac malformations (C), tracheoesophageal fistula with esophageal atresia (TE), renal dysplasia (R), and limb anomalies (L)
