# Supplementary material for: Patients with anorectal malformation and upper limb anomalies: genetic evaluation is warranted
Source: Eur J Pediatr. 2015 Oct 24;175:489–97. doi: 10.1007/s00431-015-2655-9 (PMC4799257; doi:10.1007/s00431-015-2655-9)
Supplement: Supplementary file 1 — (DOC 68 kb) [file 431_2015_2655_MOESM1_ESM.doc]

**Supplementary file for ‘Anorectal malformation patients with upper limb anomalies: genetic counseling is warranted’, European Journal of Pediatrics**

Desiree van den Hondel; Charlotte HW Wijers; Yolande van Bever; Annelies de Klein; Carlo LM Marcelis; Ivo de Blaauw; Cornelius EJ Sloots; Hanneke IJsselstijn.

Corresponding author: Hanneke IJsselstijn. E-mail: h.ijsselstijn@erasmusmc.nl

Table 4: Prevalence of genetic disorders in anorectal malformation patients with associated anomalies in different organ systems.

| **Associated anomaly** | | **Genetic disorder present** |
| --- | --- | --- |
| **Lower limb** | Yes | 13/46 (28%) |
|  | No | 53/438 (12%) |
| **Cardiac** | Yes | 34/171 (22%) |
|  | No | 32/310 (10%) |
| **CNS** | Yes | 23/104 (22%) |
|  | No | 43/377 (11%) |
| **Urogenital** | Yes | 28/298 (9%) |
|  | No | 38/183 (21%) |
| **Other GI** | Yes | 11/89 (12%) |
|  | No | 55/384 (14%) |
| **Vertebral** | Yes | 12/168 (7%) |
|  | No | 54/313 (17%) |

Abbreviations: CNS, central nervous system; GI, gastro-intestinal.
Total number of non-isolated ARM patients: n=481.

**Table 5: Correlation for associated anomalies in anorectal malformations.**

|  | | **Upper limb** | | **Lower limb** | | **Vertebral** | | **Other GI** | | **Cardiac** | | **Urogenital** | | CNS | |  | |
| --- | --- | --- | --- | --- | --- | --- | --- | --- | --- | --- | --- | --- | --- | --- | --- | --- | --- |
|  | |  |  | Yes | No | Yes | No | Yes | No | Yes | No | Yes | No | Yes | No |  | |
| **Upper limb** | Phi |  | | 8 | 35 | 21 | 22 | 19 | 24 | 26 | 17 | 26 | 17 | 8 | 35 | Yes | **Upper limb** |
|  |  |  |  | 38 | 400 | 147 | 291 | 78 | 360 | 145 | 293 | 272 | 166 | 96 | 342 | No |  |
| **Lower limb** | Phi | 0.096 | |  | | 15 | 31 | 12 | 34 | 11 | 35 | 31 | 15 | 19 | 27 | Yes | **Lower limb** |
|  |  |  |  |  |  | 153 | 282 | 85 | 350 | 160 | 275 | 267 | 168 | 85 | 350 | No |  |
| **Vertebral** | Phi | 0.091 | | 0.016 | |  | | 40 | 128 | 53 | 115 | 110 | 58 | 42 | 126 | Yes | **Vertebral** |
|  |  |  |  |  |  |  |  | 57 | 256 | 118 | 195 | 188 | 125 | 62 | 251 | No |  |
| **Other GI** | Phi | 0.188 | | 0.048 | | 0.067 | |  | | 45 | 52 | 53 | 44 | 24 | 73 | Yes | **Other GI** |
|  |  |  |  |  |  |  |  |  |  | 126 | 258 | 245 | 139 | 80 | 304 | No |  |
| **Cardiac** | Phi | 0.163 | | 0.079 | | 0.061 | | 0.114 | |  | | 85 | 86 | 33 | 138 | Yes | **Cardiac** |
|  |  |  |  |  |  |  |  |  |  |  |  | 213 | 97 | 71 | 239 | No |  |
| **Urogenital** | Phi | 0.010 | | 0.036 | | 0.053 | | 0.076 | | 0.187 | |  | | 66 | 232 | Yes | **Urogenital** |
|  |  |  |  |  |  |  |  |  |  |  |  |  |  | 38 | 145 | No |  |
| **CNS** | Phi | 0.023 | | 0.155 | | 0.060 | | 0.038 | | 0.042 | | 0.016 | |  | |  | **CNS** |
|  |  |  |  |  |  |  |  |  |  |  |  |  |  |  |  |  |  |

Phi-values are shown on the left side, and number s of patients on the right side.
The phi value is the correlation coefficient based on chi-squared analysis. It measures the association in which two anomalies tend to co-occur, regardless of occurrence of other anomalies. 0-0.25 represents poor association, 0.50 moderate association, 0.75 strong association, 1 represents complete association.
